# The dynamic behavior of Ect2 in response to DNA damage

**DOI:** 10.1038/srep24504

**Published:** 2016-04-14

**Authors:** Dan He, Jinnan Xiang, Baojie Li, Huijuan Liu

**Affiliations:** 1 Bio-X Institutes, Key Laboratory for the Genetics of Developmental and Neuropsychiatric Disorders, Ministry of Education, Shanghai Jiao Tong University, Shanghai 200240, China

## Abstract

Ect2 is a BRCT-containing guanidine exchange factor for Rho GTPases. It is essential for cytokinesis and is also involved in tumorigenesis. Since most BRCT-containing proteins are involved in DNA damage response and/or DNA repair, we tested whether Ect2 plays similar roles. We report that in primary mouse embryonic fibroblasts (MEFs), DNA damage quickly led to Ect2 relocalization to the chromatin and DNA damage foci-like structures. Ect2 knockdown did not affect foci localization of γH2AX, TopBP1, or Brca1, or activation of Atm, yet it impeded p53 Ser15 phosphorylation and activation, and resulted in defects in apoptosis and activation of S and G2/M checkpoints in response to DNA damage. These results suggest that Ect2 plays a role in DNA damage response. Interestingly, Ect2 is down-regulated at late stages of DNA damage response. Although p53 and E2F1 have been shown to regulate Ect2 transcription, DNA damage-induced Ect2 down-regulation occurred in p53−/− or Atm−/− MEFs and E2F1 knockdown cells. Instead, DNA damage-induced Ect2 down-regulation is mainly attributable to decreased protein stability. Like Ect2 knockdown, Ect2 destabilization may help the cell to recover from DNA damage response. These results suggest that Ect2 plays roles in multiple aspects of DNA damage response.

DNA is under constant attack by endogenous reactive oxygen species and exogenous genotoxic reagents including some chemotherapeutic drugs. The cell senses DNA damage and transmits the stress signals to effector molecules to cause cell cycle arrest and/or apoptosis^1^,^2^,^3^. At the center of the DNA damage response (DDR) lie the PI3 kinase-like kinases (PIKKs) including Atm and Atr^4^,^5^,^6^,^7^. Atm is activated at the sites of double stranded DNA breaks, where a large number of proteins are assembled, forming the DNA damage foci^8^. Atm phosphorylates many foci proteins, e.g., Mdc1 and Nbs1, and non-foci proteins, e.g., p53, Chk2, and Smad1^9^,^10^,^11^,^12^. p53 activation induces cell cycle arrest and/or apoptosis via p21, Bax, Puma and other target genes. This helps to maintain the integrity of the genome and prevents the accumulation of gene mutations^13^. As such, the DNA damage response is the predominant tumor suppression pathway^14^. In addition, chemotherapy and radiotherapy exert their anti-tumor effects via activating the DNA damage response.

Many proteins involved in DNA damage response contain Brca1 C terminal (BRCT) domains^15^,^16^,^17^. The BRCT domain has been shown to directly bind to phospho-peptides, especially proteins phosphorylated by Atm/Atr, other BRTC-containing proteins, DNA breaks, and poly (ADP-ribose)^15^,^17^,^18^. The interaction mediated by BRCT domains affects the localization and/or the function of these proteins. For example, the BRCT domain helps to retain 53BP1 at the DNA damage foci via interacting with Ser139 phosphorylated H2AX^19^. BRCT domains are present in 23 human proteins as a single or tandem repeats, e.g., DNA Pol I λ and μ, XRCC, Lig3, BRCA1, 53BP1, MDC1, Lig4, and TopBP1. Brca1 is a tumor suppressor whose mutations in the BRCT domain increase the risks of breast cancer and ovarian cancer^16^,^17^.

Ect2 (Epithelial cell transforming sequence 2) is a BRCT-containing protein whose function is best studied in cytokinesis^20^,^21^,^22^. It is a guanine nucleotide exchange factor (GEF) for Rho small GTPases^22^,^23^,^24^,^25^. Yet Ect2 is only one of the 25 GEFs that can activate Rho GTPases^26^. Ect2 is activated in prophase and then is relocated to the equatorial membrane^27^. The BRCT domain of Ect2 interacts with MKlp1-MgcRacGAP complex at the central spindle, where Ect2 promotes the assembly and constriction of actomyosin^28^. Furthermore, Ect2 is essential for mouse embryonic development. Ect2 ablation leads to embryonic lethality at E3.5 and Ect2−/− MEFs showed an increase in binucleated cells and a defect in cell migration^26^.

Ect2 is highly expressed in various types of human tumors^29^,^30^,^31^. Elevated levels of Ect2, especially the cytoplasmic Ect2^32^, is believed to potentiate tumorigenesis via activating small GTPases such as Rho, Rac, and Ras^23^,^31^. Ect2 expression is controlled at both transcription and post-transcriptional levels. Ect2 transcription is positively regulated by E2F1 and Cux1 in the S phase and negatively regulated by p53 under genotoxic conditions in cancer cell lines^25^,^33^. Ect2 promoter regions contain p53 binding sites, which suppresses Ect2 transcription in cooperation with protein methyltransferase^29^. Ect2 protein can be degraded right after mitosis via APC/C-mediated ubiquitination^34^.

Based on the fact that Ect2 contains a BRCT domain, we suspect that Ect2 might play a role in DNA damage response. Indeed we found that DNA damage led to localization of Ect2 to the chromatin and foci-like structures, where it partially overlapped with γH2AX. Its presence is required for p53 activation, apoptosis, and activation of S and G2/M checkpoints in response to DNA damage. At later stages of the DNA damage response, Ect2 is down-regulated owing to protein destabilization rather than Atm-p53-controlled transcription. These findings suggest that Ect2 has two functions in DDR: its initial chromatin/foci localization may assist PIKKs-mediated p53 phosphorylation and activation, and its later degradation may help the cell to recover from the DNA damage response.

## Results

### Ect2 was quickly relocalized onto the DNA damage foci-like structures

**Figure 1:**
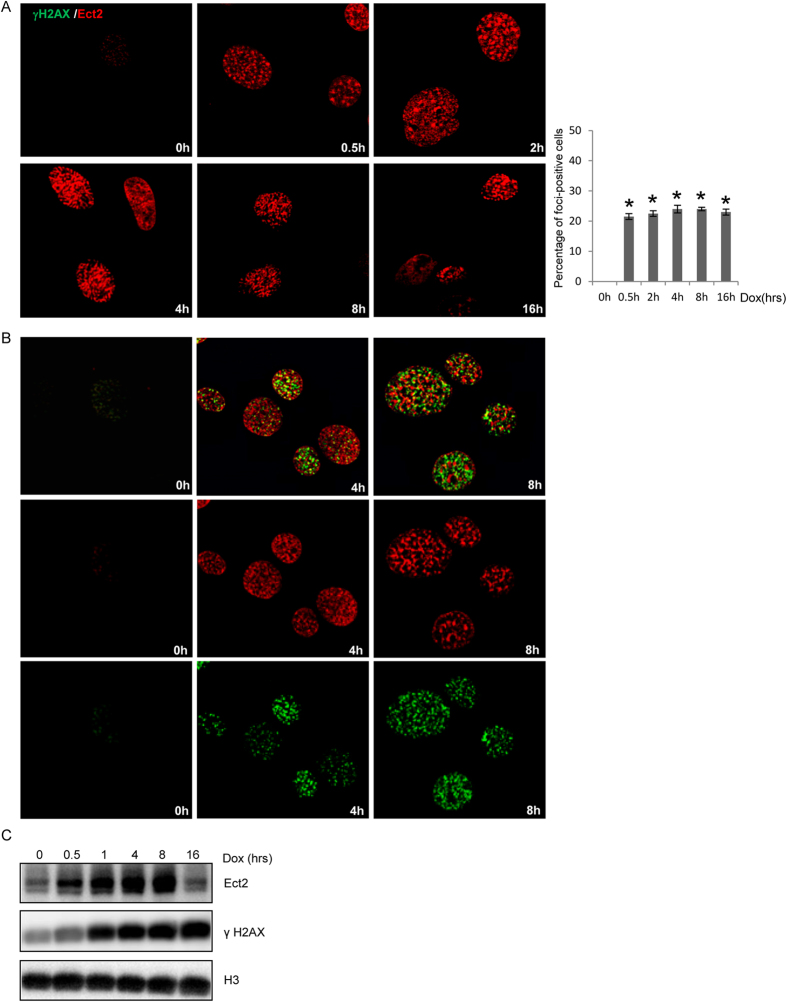
Ect2 was localized onto the chromatin and foci-like structures. (**A**) DNA damage led to quick localization of Ect2 onto the foci-like structures. Primary MEFs grown on coverslips were treated with 1 μM of Dox for 0.5, 2.0, 4.0, 8.0, or 16 hrs, which were collected, fixed, and then immuno-stained for Ect2 using Texas-Red conjugated secondary antibodies. Right panel: Percentage of cells positive for Ect2 foci. (**B**) Ect2 was partially co-localized with γH2AX on Dox-induced foci. Primary MEFs grown on coverslips were treated with 1 μM of Dox for different periods of time. The cells were collected, fixed, and then immuno-stained for Ect2 and γH2AX using Texas-Red or FITC conjugated secondary antibodies. (**C**) DNA damage resulted in chromatin association of Ect2 in MEFs. MEFs were treated with Dox for different periods of time. The cells were harvested and the chromatin fractions were isolated. Western blot analysis was used to detect the protein levels of Ect2, H3 and γH2AX. For the full-length blots see Supplementary Fig. S1C.

Most of the BRCT-containing proteins are involved in DDR and/or DNA repair and some of them are localized to DNA damage-induced nuclear foci, e.g., Mdc1, TopBP1, 53BP1, and Brca1^16^,^17^. We wanted to test whether the BRCT-containing protein Ect2 was also localized at DNA damage foci. We used primary MEFs for the study as primary cells, unlike tumor cell lines, have intact DDR^35^. We found that in response to doxorubicin (Dox), a chemotherapeutic drug that generates both double stranded and single stranded DNA breaks^36^,^37^, Ect2 was localized onto the foci-like structures as early as 30 min after Dox treatment (Fig. 1A). Moreover, ionizing radiation (IR) and teniposide, a DNA topoisomerase inhibitor, also induced Ect2 localization to the foci-like structures (Supplementary Fig. S1A and S1B).

One earliest response to DNA damage is H2AX phosphorylation by Atm/Atr/DNA-PKcs at the DNA breaks, generating γH2AX positive foci^8^,^38^. We found that Ect2 showed partial overlapping with γH2AX in some of the foci-like structures (Fig. 1B). We also tested whether inhibition of Atm/Atr with caffeine had any effect on Ect2 foci formation, and found that pre-treatment of cells with caffeine only modestly reduced the number of Ect2 positive foci (data not shown), suggesting that Atm/Atr activity has a minor effect on Ect2 assembly at the foci.

To validate the foci association of Ect2, we tested whether Ect2 was bound to the chromatin after DNA damage in MEFs. We found that chromatin-bound Ect2 gradually went up with time in response to Dox and it then went down 16 hrs after Dox treatment (Fig. 1C and Supplementary Fig. S1C). These results, taken together, suggest that Ect2 is localized to the chromatin and foci-like structures in response to DNA damage.

### Ect2 depletion did not alter foci formation for γH2AX, TopBP1, or Brca1

**Figure 2:**
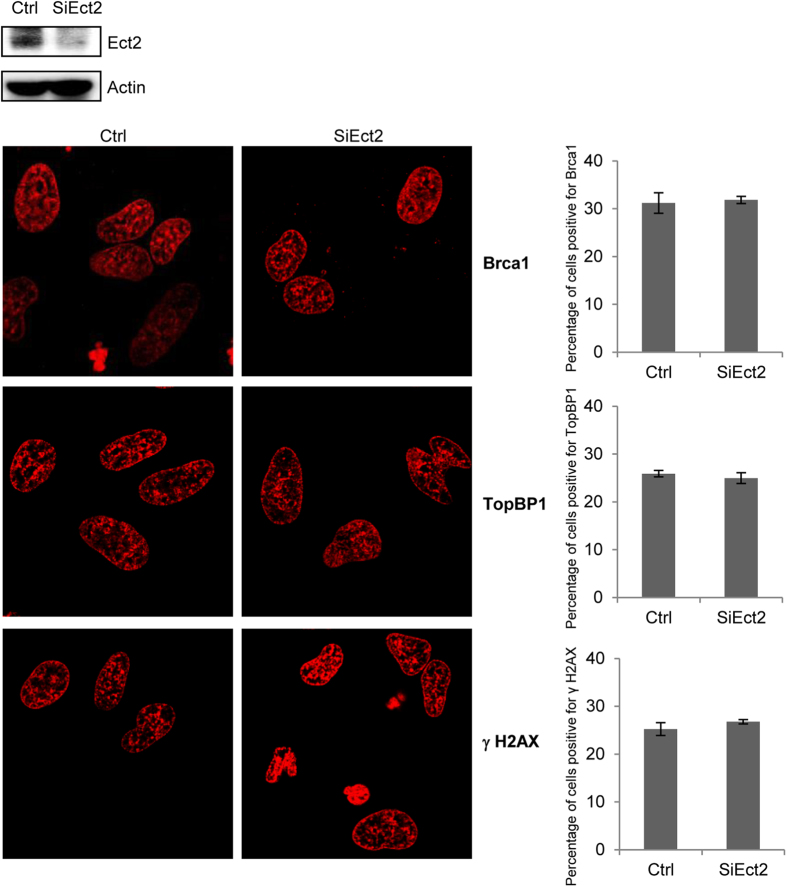
Depletion of Ect2 did not significantly alter the foci formation of TopBP1, Brca1, or γH2AX. Primary MEFs grown on coverslips were transfected with control or Ect2 siRNA and then were treated with 1 μM of Dox for 4.0 hrs. The cells were collected, fixed, and the immuno-stained for Brca1, TopBP1, or γH2AX using Texas-Red conjugated secondary antibodies. Upper panel: WB showing knockdown of Ect2. For the full-length blots see Supplementary Fig. S2. Right panel: quantitation data showing the percentage of foci-positive cells. Right panel: percentage of foci-positive cells.

The nuclear foci are believed to be centers of signal propagation and DNA repair, where dozens of proteins are assembled in a spatiotemporal manner^8^. Protein-protein interaction mediated by BRCT domains has been shown to play an important role in recruiting many proteins to the DNA damage foci. To test whether Ect2 plays a role in the recruitment of other BRCT-containing proteins, we knocked down Ect2 in primary MEFs with siRNA (Fig. 2 and Supplementary Fig. S2A), and treated the cells with Dox. It was found that Ect2 knockdown did not significantly altered recruitment of BRCT-containing proteins Brca1 and TopBP1 onto the foci (Fig. 2), nor did it affect foci assembly of γH2AX (Fig. 2). These results indicate that unlike some of the foci proteins, Ect2 is not required for recruitment of Brca1, TopBP1, or γH2AX to the DNA damage foci. Note that Ect2 knockdown only led to accumulation of a limited number of polyploidy cells (Supplementary Fig. S2B).

### A role for Ect2 in activation of p53 in response to DNA damage

**Figure 3:**
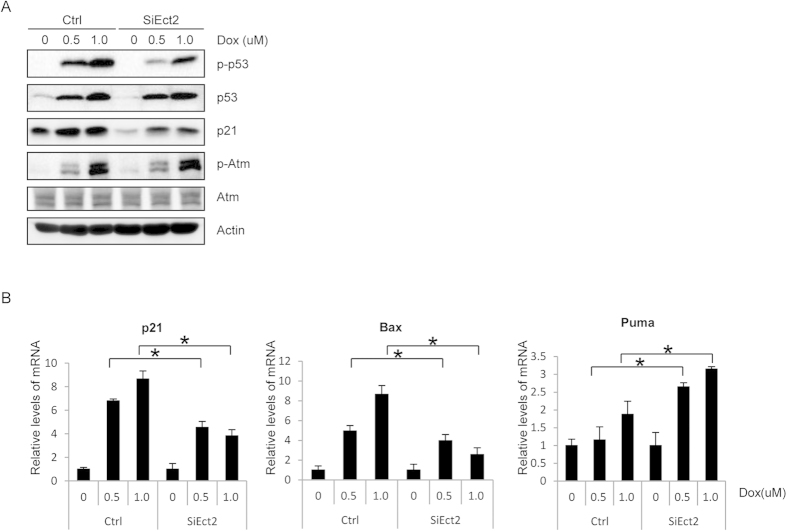
Ect2 is required for p53 Ser15 phosphorylation and activation. (**A**) Western blot shows that knockdown of Ect2 decreased Dox-induced p53 Ser15 phosphorylation and p21 expression at the basal levels and in response to DNA damage. For the full-length blots see Supplementary Fig. S3. For Ect2 knockdown, see Fig. 2 upper panel. (**B**) Quantitative PCR assay shows that Ect2 knockdown inhibited the expression of p21 and Bax in primary MEFs. Primary MEFs were transfected with control or Ect2 siRNA for 48 hrs and then treated with 0.5 or 1 μM of Dox for 8 hrs, which were collected. Total RNA was isolated from these cells and reverse transcribed. Quantitative PCR was used to determine the mRNA levels of p21, Bax, Puma, and Actin. N = 3.

At the DNA damage foci, Atm and Atr phosphorylate effector molecules such as p53 and Chk2, which shuttle on and off the DNA damage foci^6^. We then analyzed the activation of p53, the best-studied substrate of Atm/Atr in DDR. We found that in Ect2 knockdown MEFs, p53 phosphorylation on S15 was significantly decreased compared to control cells, although the p53 protein levels were not significantly altered except at the basal levels (Fig. 3A and Supplementary Fig. S3). Moreover, we found that the induction of p21 (Fig. 3A and Supplementary Fig. S3), one of the target genes of p53 in DDR, was also impeded in Ect2 knockdown cells at the basal level and in response to DNA damage, which may be caused by the decrease in p53, p53 phosphorylation, or both.

We also analyzed the mRNA levels of p21, Puma, and Bax, the p53 target genes, in Ect2 knockdown and control cells with quantitative PCR. It was found that the mRNA levels of p21, Puma, and Bax were up-regulated in normal cells in response to DNA damage, and Ect2 knockdown significantly inhibited the increase in the mRNA levels of p21 and Bax but not Puma (Fig. 3B). These results demonstrate, for the first time, that Ect2 plays a role in p53 activation in DDR and that Ect2 may also regulate Puma expression via p53-independent fashions.

### Ect2 was not required for Atm activation in DNA damage response

Based on our observation that Ect2 is localized onto the nuclear foci-like structures and is required for optimal p53 Ser15 phosphorylation in response to DNA damage, we suspected that Ect2 might be involved in Atm activation. However, western blot analysis showed that Ect2 knockdown did not significantly affect the activation of Atm, justified by unchanged levels of Atm phosphorylation at S1981 (Fig. 3A and Supplementary Fig. S3). We could not exclude the possibility that Ect2 might be involved in the activation of other PIKKs. Ect2 may act as an adaptor to assist Atm/Atr-mediated phosphorylation of p53 or regulate protein complex formation at the DNA damage foci to assist p53 phosphorylation, which warrants further investigation.

### Knockdown of Ect2 led to a defect in Dox-induced apoptosis

**Figure 4:**
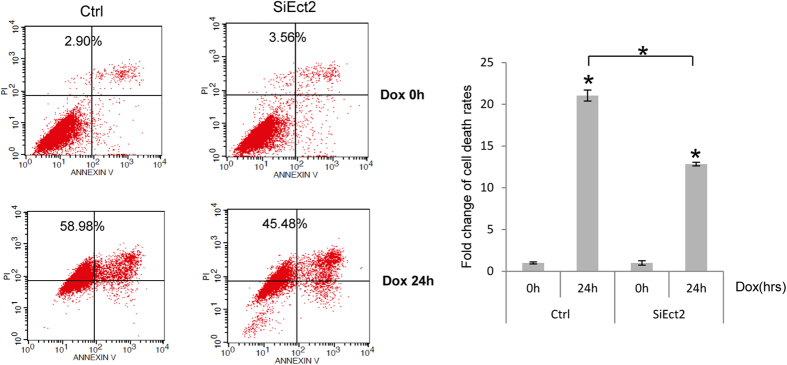
Knockdown of Ect2 impaired Dox-induced apoptosis in MEFs. Primary MEFs were transfected with control or Ect2 siRNA for 48 hrs and then treated with 1 μM of Dox for 24 or 48 hrs, which were collected. Cell death rates were determined by Annexin V-FITC/PI apoptosis assay kit (Right panel). Left panel: comparison of Dox-induced cell death rates in Ect2 knockdown and control MEFs. To determine the effects of Ect2 knockdown on cell death rates, the basal cell death rates of Ect2 knockdown and control cells were set at 1. N = 3.

The cells sense DNA damage by Atm and other PIKKs, which transmit the signals to the effector molecules, e.g., p53, to cause cell cycle arrest or apoptosis^10^,^13^. Since Ect2 is involved in p53 activation, we tested whether it played a role in DNA damage-induced apoptosis. We first knocked down Ect2 with siRNA in primary MEFs and found that Ect2 knockdown did not significantly affect cell survival, judged by Wst-1 assay results (Fig. 2 and Supplementary Fig. S4). We then treated these cells and control cells with Dox for 24 hrs. The cells were then harvested and cell death rates were measured by Annexin V-FITC/PI apoptosis assay kit. Dox induced an increase in the number of cells positive for PI/Annexin or PI in control MEF cultures and Ect2 knockdown significantly impeded Dox-induced cell death rates (Fig. 4). These results suggest that Ect2 plays a role in DNA damage-induced cell death.

### Knockdown of Ect2 led to defects in S and G2/M cell cycle arrest

**Figure 5:**
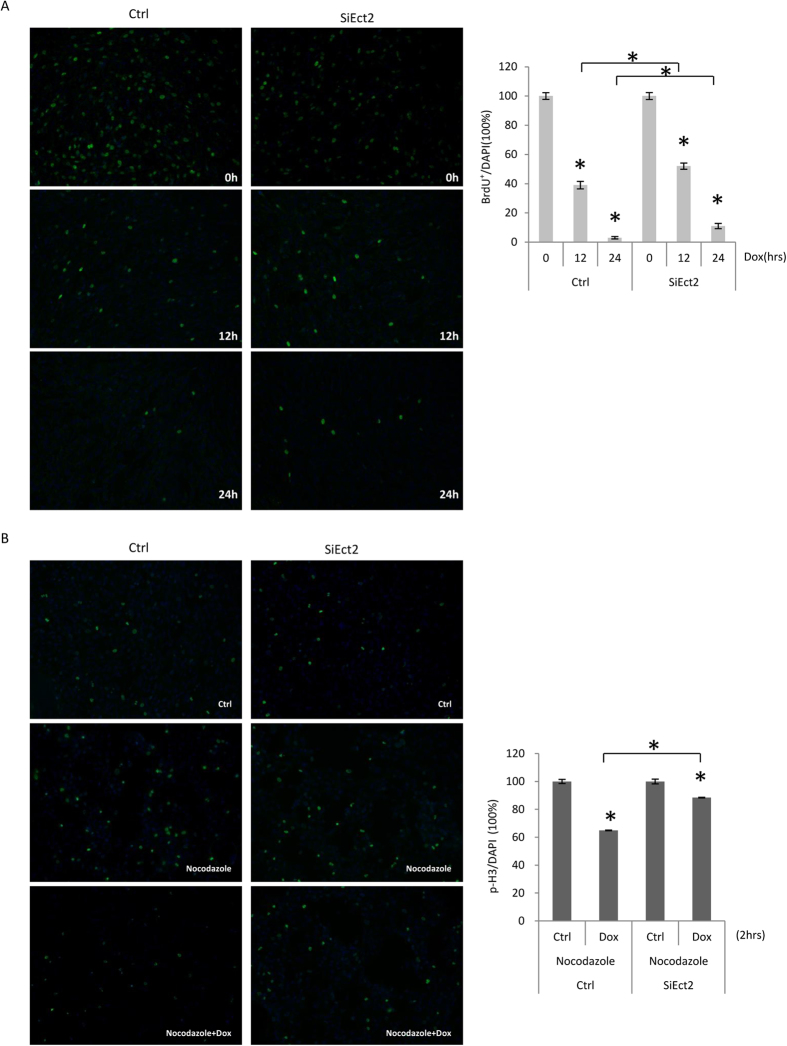
Knockdown of Ect2 impaired activation of the S and G2/M phase checkpoints. (**A**) Primary MEFs were transfected with control or Ect2 siRNA for 48 hrs and then treated with 1 μM of Dox for 12 or 24 hrs, followed by incubation with BrdU for 1 hr. The cells were collected and stained for BrdU by immunofluorescent staining. Right panel: quantitation data. The BrdU^+^/nucleus values of untreated cells were set at 100%. N = 3. (**B**) Primary MEFs were transfected with control or Ect2 siRNA for 48 hrs and then treated with nocodazole or nocodazole plus 1 μM of Dox for 2 hrs. The cells were then collected and stained for p-H3. Right panel: quantitation data. The p-H3/nucleus values of untreated cells were set at 100%. N = 3. For longer time points, see Supplementary Fig. S5.

We then tested whether Ect2 plays a role in DNA damage-induced cell cycle arrest. We treated MEFs transfected with control or Ect2 siRNA with Dox for 12 or 24 hours, followed by BrdU labeling for 1 hr. The cells were then harvested and analyzed for the activation of cell cycle checkpoints. Staining for S phase cells with BrdU antibodies revealed that Dox treatment induced a decrease in S phase cells in normal MEF cultures (Fig. 5A), while Ect2 knockdown cells showed a greater number of S phase cells than that in control cells in response to Dox treatment (Fig. 5A), suggesting that Ect2 is required for optimal activation of the S phase checkpoint, consistent with the well-established role for p53 activation in regulating cell cycle entry.

We then looked at DNA damage-induced cell cycle arrest at the G2/M phase. It is known that DNA damage delays or prevents cells from entering the mitosis phase. We adopted a method that used nocodazole treatment to arrest the cells at the prometaphase, which was detected by phospho-Histone 3 (p-H3). We treated the cells with nocodazole alone or with Dox for 0, 2, 4, or 8 hrs and p-H3 positive cells were counted. It was found that Dox, like other genotoxic drugs, impaired cell entry into the prometaphase, manifested by a reduction in the number of cells positive for p-H3. Ect2 knockdown cells showed an increase in cells positive for p-H3 compared to cells transfected with control siRNA (Fig. 5B and Supplementary Fig. S5A and S5B). These results indicate that Ect2 is required for proper G2/M checkpoint activation.

### Ect2 was down-regulated at late stages of DDR

**Figure 6:**
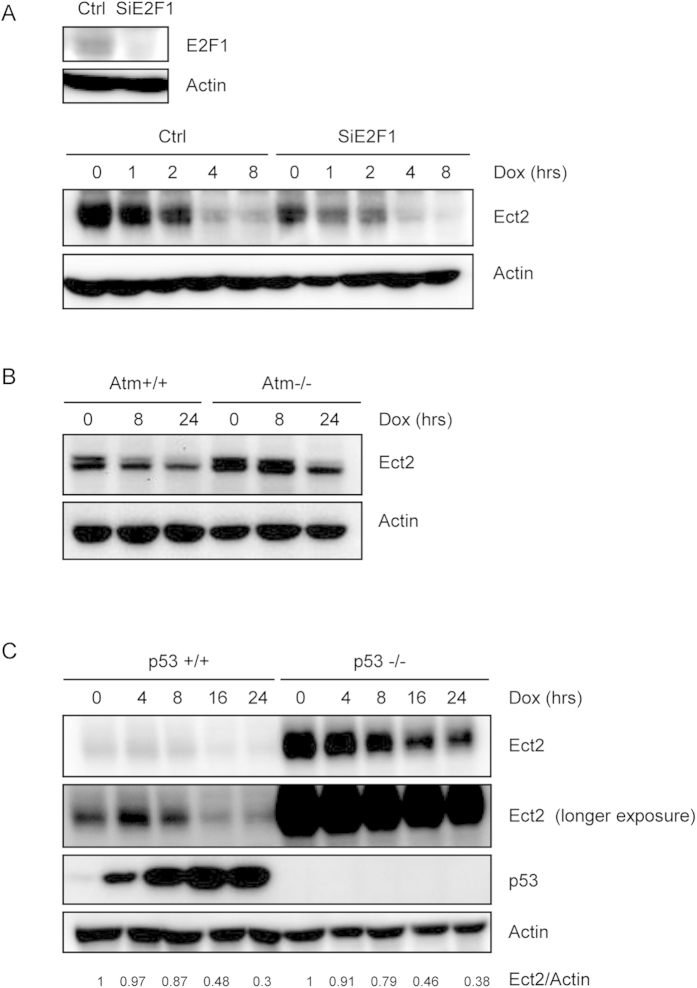
Ect2 was down-regulated at the later stages of DNA damage response independent of Atm, p53, or E2F1. (**A**) MEFs with E2F1 knockdown still showed Ect2 down-regulation in response to DNA damage. Primary MEFs were transfected with control or E2F1 siRNA for 48 hrs and then treated with 1 μM of Dox for different periods of time. Western blot was used to determine the protein levels of Ect2. Upper panel: WB showing knockdown of E2F1. For the full-length blots see Supplementary Fig. S6. (**B**) MEFs deficient for Atm still showed Ect2 down-regulation in response to DNA damage. Primary Atm−/− and control MEFs were treated with 1 μM of Dox for different periods of time. Western blot was used to determine the protein levels of Ect2. For the full-length blots see Supplementary Fig. S7. (**C**) MEFs deficient for p53 still showed Ect2 down-regulation in response to DNA damage. Primary p53−/− and control MEFs were treated with 1 μM of Dox for different periods of time. Western blot was used to determine the protein levels of Ect2. For the full-length blots see Supplementary Fig. S8.

We also noticed that primary MEFs showed a decrease in the protein levels of Ect2 8 hrs after Dox treatment (Fig. 6A and Supplementary Fig. S6). Previous studies also reported that DNA damage down-regulated Ect2 in cancer cell lines, which is believed to cause G1 cell cycle arrest^29^. Yet we showed here that Ect2 knockdown also affected G2/M checkpoint as well as cell death in primary MEFs (Figs 4 and 5). Previous studies have shown that Ect2 expression can be regulated at the transcriptional level by E2F1 and p53^33^,^39^. p53 binds to the Ect2 promoter and suppress Ect2 transcription, whereas E2F1 promotes Ect2 transcription in S phase. We then determined whether p53 or E2F1 was involved in DNA damage-induced Ect2 down-regulation. We first knocked down E2F1 with siRNA in MEFs and then stressed the cells with Dox for different periods of time. Although E2F1 knockdown decreased the basal levels of Ect2, Dox treatment still induced Ect2 down-regulation (Fig. 6A and Supplementary Fig. S6), suggesting that E2F1 is not involved in DNA damage-induced Ect2 down-regulation.

We then used Atm−/− and p53−/− primary MEFs to test the possible involvement of the Atm-p53 pathway in Ect2 down-regulation under genotoxic conditions. We found that while Atm or p53 deficiency increased the basal levels of Ect2, Dox still induced Ect2 down-regulation in primary MEFs deficient for Atm or p53, although to slightly reduced extents compared to control cells (Fig. 6B,C and Supplementary Figs S7 and S8). These results suggest that the Atm-p53 pathway is not essential for Ect2 down-regulation in DDR. This is in contrast to the previous findings obtained from cancer cell lines^29^.

### DNA damage promoted Ect2 destabilization at late stages of DDR

**Figure 7:**
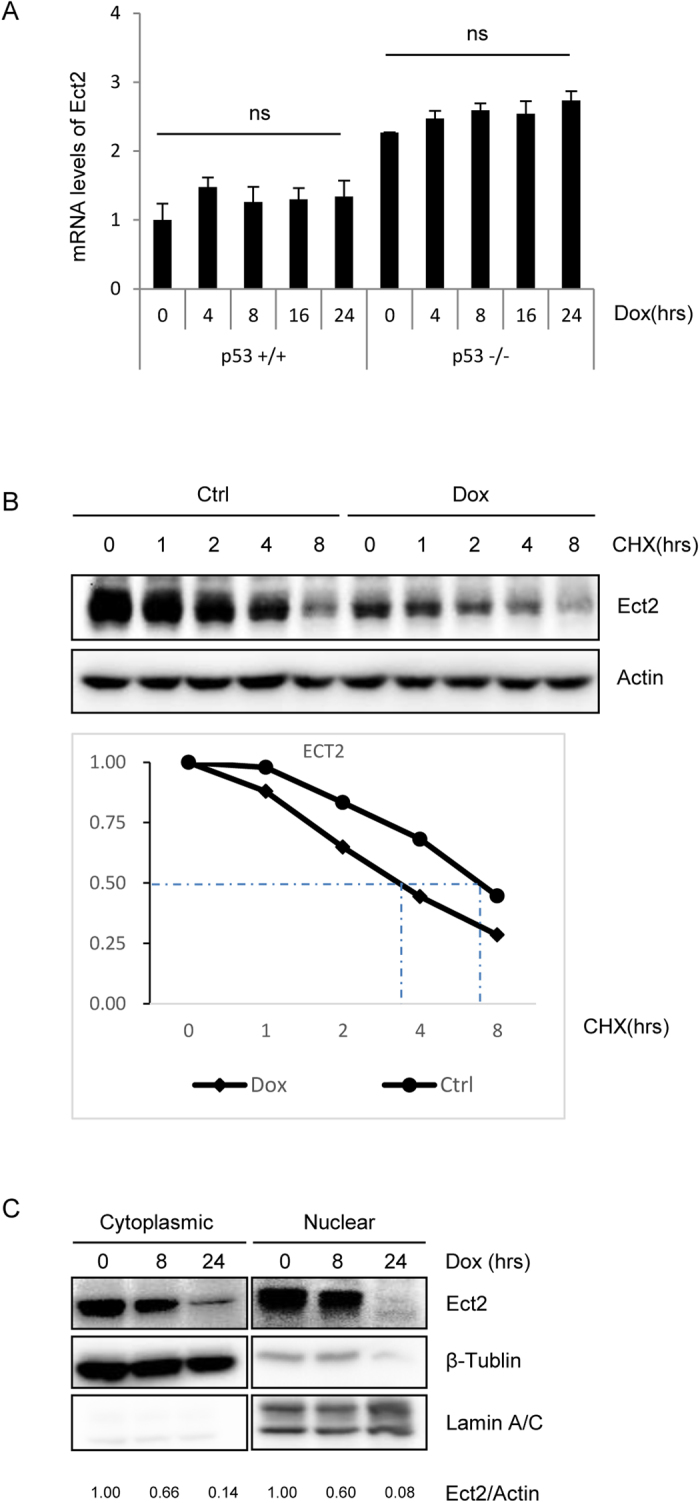
DNA damage led to accelerated Ect2 protein degradation in MEFs. (**A**) DNA damage-induced Ect2 down-regulation did not occur at the mRNA levels. Primary p53−/− and control MEFs were treated with 1 μM of Dox for different periods of time. Total RNA was isolated from the cells and quantitative PCR results showed that the mRNA levels of Ect2 were not decreased by Dox treatment in WT or p53−/− MEFs. N = 3. (**B**) Ect2 protein degradation was enhanced in MEFs in response to Dox treatment. Primary MEFs were treated with 1 μM of Dox for 2 hrs and then 10 μg/ml cycloheximide was added. The cells were harvested at different periods of time after cycloheximide addition. Western blot was used to determine the protein levels of Ect2. For the full-length blots see Supplementary Fig. S9. Bottom panel: quantitation data. (**C**) Both cytoplasmic and nuclear Ect2 molecules were degraded in response to Dox treatment. Primary MEFs were treated with 1 μM of Dox for 8 or 24 hrs. The cells were harvested and were separated into the cytoplasmic and nucleus fractions. Western blot was used to determine the protein levels of Ect2 in these two fractions. For the full-length blots see Supplementary Fig. S10.

Interestingly, quantitative PCR analysis revealed that Dox did not alter the levels of Ect2 mRNA levels in primary WT or p53−/− MEFs (Fig. 7A), suggesting that DNA damage may suppress Ect2 expression at the post-translational levels in primary cells. This is inconsistent with the results obtained from cancer cell lines^29^. We then compared the degradation of Ect2 protein in normal MEFs in the presence or absence of DNA damage, and found that Ect2 protein was degraded at an increased rate, with the halflife down from 7.1 hrs to 3.3 hrs, in response to DNA damage (Fig. 7B and Supplementary Fig. S9). These results suggest that DNA damage induces Ect2 down-regulation via accelerated degradation rather than suppressed transcription.

Ect2 is known to be localized in both cytoplasm and nucleus. Its oncogenic activity is believed to be mediated by cytoplasm-localized Ect2, where it activates PKCi, Ras, and other small GTPases to transform cells^31^. On the other hand, we showed that nucleus-localized Ect2 molecules were involved in DNA damage response and p53 activation. We then wanted to test whether DNA damage affected the distribution of Ect2 protein. Primary MEFs were treated with Dox for different periods of time, collected, and separated into cytoplasm and nuclear fractions. Western blot analysis showed that Ect2 molecules were distributed in both cytoplasm and nucleus at the basal level, and Dox treatment reduced Ect2 protein levels to similar extents in the cytoplasm and nucleus (Fig. 7C and Supplementary Fig. S10), suggesting that Ect2 is similarly degraded in the cytoplasm and nucleus under genotoxic stress.

## Discussion

Previous studies have established a critical role for Ect2 in cytokinesis and tumorigenesis^21^,^31^,^40^. Although it has been long known that Ect2 has a BRCT domain, its roles in DDR have not been fully explored. This study revealed that Ect2 might play multiple roles in DNA damage response, one of the prominent tumor suppressing pathways. At the early stages of DNA damage response, Ect2 is localized to the chromatin and DNA damage foci-like structures. It is worth noting that unlike other early foci proteins, Ect2 only shows partial overlapping with γH2AX and it is not involved in foci assembly of H2AX, Brca1, or TopBP1, or Atm activation. That being said, Ect2 appears to play a role in PIKKs-mediated p53 activation. Ect2 knockdown leads to a decrease in Dox-induced phosphorylation of p53 on Ser15 and the expression of p53 target genes including p21 and Bax. More importantly, Ect2 is required for DNA damage-induced cell death, S phase checkpoint activation, and G2/M checkpoint activation in primary MEFs. These biochemical and function data suggest that Ect2, a BRCT containing protein, plays an important role in DNA damage response. Interestingly, Ect2 is down-regulated at later stages of DNA damage response. This, like Ect2 knockdown, may act to help the cell to recover from DNA damage response, which warrants further investigation.

How Ect2 is recruited to the chromatin and foci-like structures and how it modulates PIKK-mediated p53 phosphorylation and activation remain unclear and warrant further investigation. It is possible that the BRCT domain of Ect2 may interact with phospho-peptide sequences of the foci proteins that are phosphorylated by Atm. Alternatively, the BRCT domain of Ect2 may interact with BRCT domains of other foci proteins, e.g., Brca1 and TopBP1. It will be interesting to test whether Ect2 itself is a phosphorylation substrate of Atm. It is possible that Ect2, by interacting with other foci proteins and modulating the protein complex formation at the foci, may regulate Atm-mediated p53 phosphorylation and activation. This is in contrast to several other foci proteins, e.g., Mdc1 and Nbs1, which have been shown to be required for Atm activation at the foci^8^.

It is generally believed that Ect2 is an oncogene. It is highly expressed in many types of human tumors^31^, and it is believed that cytoplasm-localized Ect2 can activate oncogenic molecules such as Rho, Rac, and Ras to transform cells^41^,^42^. However, our present study provides evidence that Ect2 may have tumor suppression activities, at least under genotoxic stress conditions, a major driving force of tumorigenesis. Ect2 helps to activate the tumor suppressor p53 and is required for execution of apoptosis and proper activation of the S and G2/M checkpoints in DNA damage response. This helps to maintain genome integrity and prevent tumorigenesis. Moreover, DNA damage-induced Ect2 down-regulation may impair cytokinesis and lead to formation of binucleated cells or polyploidy cells. Aneuploidy is a major feature of cancer cells and it can cause further DNA mutations, which is believed to promote tumorigenesis^43^. Thus down-regulation of Ect2 is associated with an increased risk for tumorigenesis. Our conclusion is consistent with the findings that Ect2 inhibits activation of the oncogenic Wnt-β-Catenin pathway, likely at a step downstream of β-Catenin destabilization^44^. Taken together, Ect2 may have oncogenic or tumor suppressive activities dependent on the cell contexts and the growth and stress conditions.

While the oncogenic activity is believed to be mediated by cytoplasmic Ect2, we show here that the tumor suppression activity may be mediated by nuclear Ect2, especially chromatin-bound Ect2. The location-dependent functions of Ect2 are reminiscent of c-Abl, another proto-oncogene product that is implicated in DNA damage response and aging^45^,^46^,^47^,^48^. c-Abl encodes a non-receptor tyrosine kinase. Fusion of c-Abl with BCR results in a constitutively active kinase, which is oncogenic and underlies the development of 95% of the chronic myeloid leukemia cases. The BCR-ABL protein is exclusively localized in the cytoplasm^49^. On the other hand, c-Abl can be found in the nucleus in addition to the cytoplasm. The nuclear c-Abl has been shown to facilitate the activation of Atm and p53 in DNA damage response, thus helping to maintain genome integrity and prevent tumor formation^50^.

Cumulative evidence indicates that Ect2 expression is actively and tightly regulated at both transcriptional and post-translational levels. While p53 can suppress Ect2 transcription by binding to the Ect2 promoter, E2F1 has been shown to promote Ect2 transcription^29^,^39^. In normal cells, Ect2 is synthesized in the S phase, likely via E2F1-mediated transactivation, with the Ect2 protein levels reaching the maximal levels in the G2/M phase. Ect2 levels quickly go down after cytokinesis, which may be attributable to Ect2 degradation^34^. In addition, Ect2 levels are increased in a large portion of human tumor cells^31^. This may be owing to loss-of-functional mutations of p53 in the tumor samples. E2F1, which is highly expressed in tumor cells, may also contribute to Ect2 up-regulation. We show here that at late stages of DNA damage response, normal cells down-regulate the levels of Ect2, which may help the cell to recover from the DNA damage response. Previous studies have shown that DNA damage represses Ect2 transcription via p53 in cancer cell lines^29^, and it is known that p53 is greatly up-regulated in normal cells. It is thus reasonable to predict that down-regulation of Ect2 in late stages of DNA damage response is caused by elevated and activated p53. However, we find that this is not the case. DNA damage-induced Ect2 down-regulation was not associated with a decrease in the mRNA levels of Ect2 in normal MEFs and moreover, MEFs deficient for Atm or p53 still showed down-regulation of Ect2 in response to DNA damage. Instead, normal cells down-regulate the levels of Ect2 at later stages of DDR mainly via enhanced protein degradation.

In summary, we have discovered previously unidentified functions for the BRCT domain-containing protein Ect2 in DNA damage response. Ect2 is located to the chromatin and DNA damage foci-like structures and it facilitates PIKK-mediated phosphorylation of p53 on Ser15, the execution of apoptosis, and the activation of S and G2/M checkpoints. Later in DDR, Ect2 is down-regulated via protein destabilization, which may help the cell to recover from DDR. This study thus uncovers dynamic behaviors of Ect2 in cell response to DNA damage.

## Materials and Methods

### Ethics statement

Animal experimentation in this study was carried out in accordance with recommendations in the National Research Council Guide for Care and Use of Laboratory Animals, with the protocols approved by the Institutional Animal Care and Use Committee of Shanghai, China [SYXK(SH)2011-0112]. The mice, including normal C57BL/6 mice, p53+/− mice, and Atm+/− mice, were maintained in SPF mouse facility of Shanghai Jiao Tong University.

### Isolation and culture of primary MEFs

The primary MEFs were derived from C57BL/6 mice. Day 13.5 embryos, with the heads and organs dissected away, were rinsed twice in PBS, minced in 500 μl of 0.25% trypsin, sheared in a 22-gauge syringe 3 times, and then incubated at 37 °C for 15–20 min. Cells were re-suspended in DMEM supplemented with 10% FBS, and plated in 60-mm dishes. The next day, dead cells and debris were removed and cells were refed with fresh media. Cells obtained at this stage were considered to be of passage zero (P0). Cells were cultured to P2 and used for experiments or frozen for future use.

### Immunofluorescent histochemistry

MEFs cultured on cover slips were washed with PBS twice and fixed in 4% PFA. The cells were then permeabilized by 0.1% Triton X in PBS for 30 min at room temperature, and blocked with 1% BSA for 30 min, and incubated with primary antibodies overnight at 4 °C. Then the slides were washed by PBS, followed by secondary antibody incubation for 60 min at 37 °C. The slides were then mounted and observed under confocal microscope. Antibody against Ect2 was purchased from Abcam(4863) and Santa Cruz Biotechnology (SC1005). Antibody against TopBP1 was purchased from BD Bioscience (611875). Antibody against Brac1 was purchased from Cell Signaling Technology. Antibody against H2AX was purchased from Bethyl (A300-081A).

### Ect2 and E2F1 knockdown

Silencer pre-designed siRNA targeting mouse ECT2 was purchased from Thermo Scientific (160638), and pre-designed siRNA targeting mouse E2F1 was purchased from Thermo Scientific (160618), which were transfected into MEFs following the manufacturer’s protocol. Knockdown efficiency was determined by immune blot.

### Western blot analysis

Cells were lysed in RIPA buffer (Beyotime) containing 1 mM PMSF and 1 μg/ml aprotonin, leupeptin, and pepstatin. The protein concentration was determined by a Bio-Rad assay. Proteins were resolved by SDS-PAGE and transferred to polyvinylidenedifluoride membranes (Millipore). Antibody against H3 was purchased from Abgent (AP50907). Antibody against H2AX was purchased from Bethyl (A300-081A). Antibodies against p-p53(Ser15)(9284), p-Atm(S1981)(5883), p53(1c12) (2524) were purchased from Cell Signaling Technology. Antibody against Atm was purchased from Genetex(GTX70103). Antibody against p21 was purchased from BD Bioscience(556430). Antibodies against Ect2-c20 (SC1005), E2F1(SC193) and β-actin (SC81178) were purchased from Santa Cruz Biotechnology. The protein bands were quantitated using the software provided by FluoChem M system (Protein Simple).

### Quantitative PCR

Total RNA was extracted from cells with TRIzol reagent (Invitrogen) following the manufacturer’s protocol. Complementary DNAs were synthesized with 0.5 μg of total RNA using Transcriptor First Strand cDNA Synthesis Kit (Roche). The detection and quantification of target mRNA were performed with real-time PCR, which were normalized to the levels of β-actin. Real-time PCR was carried out using the Applied Biosystems 7500 system.

### Annexin V-FITC/PI apoptosis assay

Annexin V-FITC/PI apoptosis detection kit was purchased from Vazyme (A211-01). 1 × 10^6^ cells were seeded on 60-mm dishes and cultured overnight. The cells were treated with 1 μM Dox for 24 hours, harvested by 0.25% trypsin digestion, re-suspended in 1ml cold PBS. The cells were pelleted by centrifugation, resuspended with 100 μl binding-buffer. We then added 5 μl Annexin V-FITC and 5 μl PI staining solution and incubated the cells for 15 minutes at room temperature. The cells were then mixed with 400 μl binding-buffer and analyzed by flow cytometry (BD bioscience).

### Cell survival assay

The assay was carried out as previously described^51^. Briefly, to measure cell survival rates after Ect2 knockdown, 10^4^ cells that were transfected with Ect2 or control siRNA for 24 hrs were seeded in 96-well plate and cultured for 48 hrs. The water-soluble tetrazolium salt (WST-1) was added to each well, and incubated for 1 h at 37 °C. The absorbance was measured against a control using microplate reader at 430 nm.

### BrdU staining

1 × 10^5^ cells were seeded on cover slips in 12-well dishes and cultured overnight. Cells were treated with 1 μM Dox for 12 or 24 hours. Before being harvested, the cells were treated by 10 μM BrdU (Roche) for 1 hr. Then the slides were washed with PBS twice, fixed in 4% PFA, permeabilized by 0.1% Triton X in PBS for 30 min at room temperature. The cells were then treated with 2N HCl, washed with PBS, and neutralized with 0.3M sodium tetraborate. After being washed by PBS for three times, the slides were blocked with 1% BSA for 30 min, incubated with primary antibody against BrdU (Roche) overnight at 4 °C, washed by PBS, followed by secondary antibody incubation for 60 min at 37 °C. The slides were then mounted and observed under microscope.

### p-H3 staining

Cells were treated with 100 nM Nocodazole alone or 100 nM Nocodazole and 1 μM Dox for 0, 2, 4, 8 hours, followed by immunofluorescent histochemistry. Antibody against p-H3(9701) was purchased from Cell Signaling Technology.

### Cell fractionation experiments

To separate cytoplasmic and nuclear fractions, 1 × 10^6^ cells were seeded on 60-mm dishes and cultured overnight and then treated with 1 μM Dox for 8 or 24 hours. Cytoplasmic and nuclear fractions were obtained using NE-PER nuclear and cytoplasmic extraction reagents (78833) purchased from Thermo Scientific, following the manufacturer’s protocol. To obtain chromatin fractions, 1 × 10^6^ cells were seeded on 60-mm dishes and cultured overnight and then treated with 1 μM Dox for 0, 0.5, 1, 4, 8, 16 hours. Chromatin fractions were obtained using Subcellular Protein Fractionation Kit purchased by Thermo Scientific (78840), following the manufacturer’s protocol.

### Protein stability assay

Briefly, 1 × 10^6^ cells were seeded on 60-mm dishes and cultured overnight. MEFs were pre-treated with 1 μM Dox for 2 hours, then with Cycloheximide (CHX 10 μg/ml). The cells were harvested at 0, 1, 2, 4, 8 hours, which were used to perform western blot to determine the Ect2 protein levels.

### Statistical analysis

Data are given as mean ± standard division (SD) of results from more than three different samples in each experiment. Differences between two groups were measured by the student’s *t*-test. A *p* value less than 0.05 is defined as statistically significant difference.

## Additional Information

**How to cite this article**: He, D. *et al.* The dynamic behavior of Ect2 in response to DNA damage. *Sci. Rep.* **6**, 24504; doi: 10.1038/srep24504 (2016).

## Supplementary Material

Supplementary Information
