# Supplementary material for: The dynamic behavior of Ect2 in response to DNA damage
Source: Sci Rep. 2016 Apr 14;6:24504. doi: 10.1038/srep24504 (PMC4830932; doi:10.1038/srep24504)

Supplementary information

The dynamic behavior of Ect2 in response to DNA damage

Dan He, Jinnan Xiang, Baojie Li*,
and Huijuan Liu*

Supplementary Figures

Figure S1. IR (10Gy) (A) and Teniposide (2μM) (B) induced relocalization of Ect2 to foci-like structures. C. Full length blots for Fig.1C.

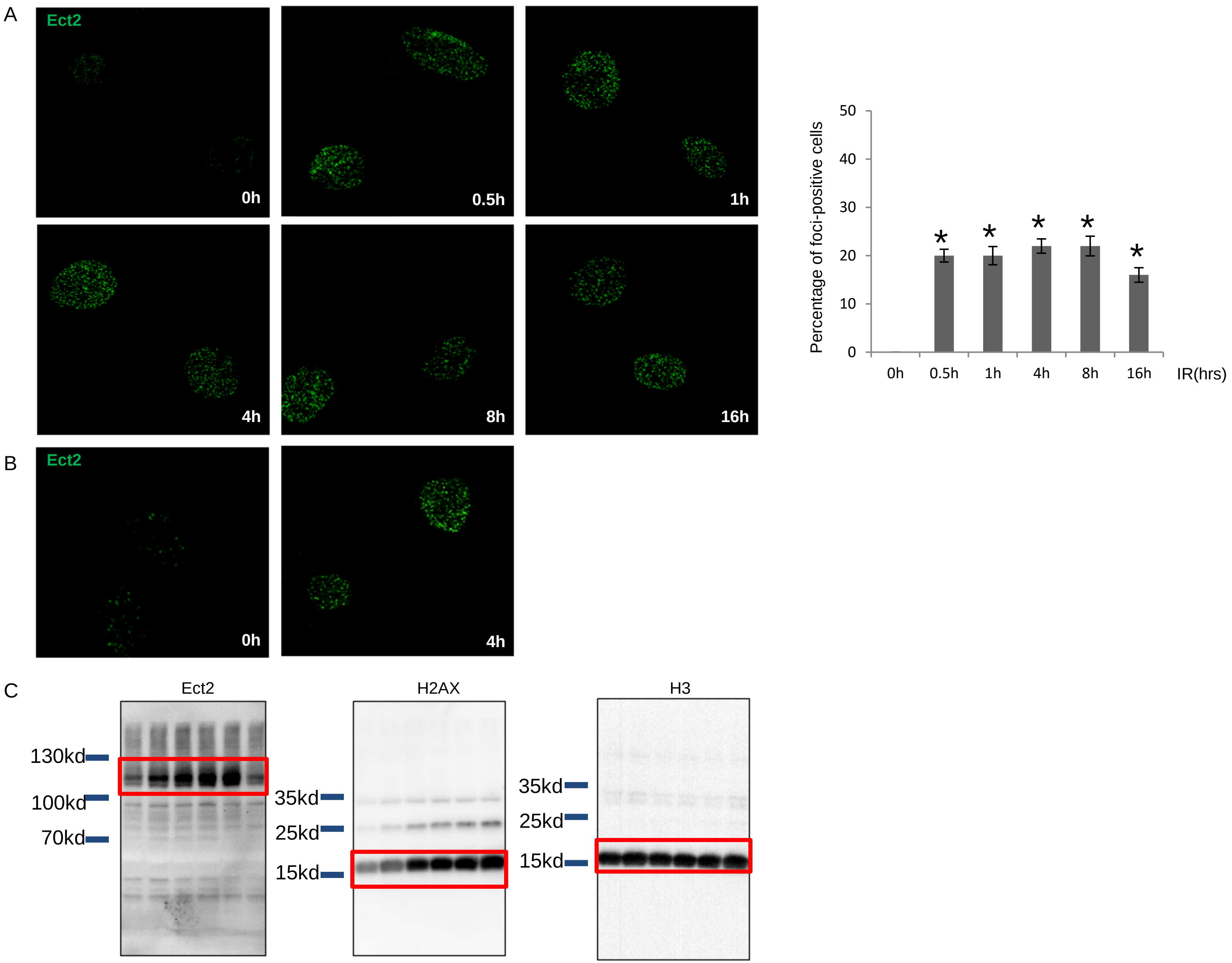

Supplementary Figures

Figure S2. A. The full length blots for Fig.2. B. FACS analysis showed knockdown of Ect2 led to polyploidy cell formation.

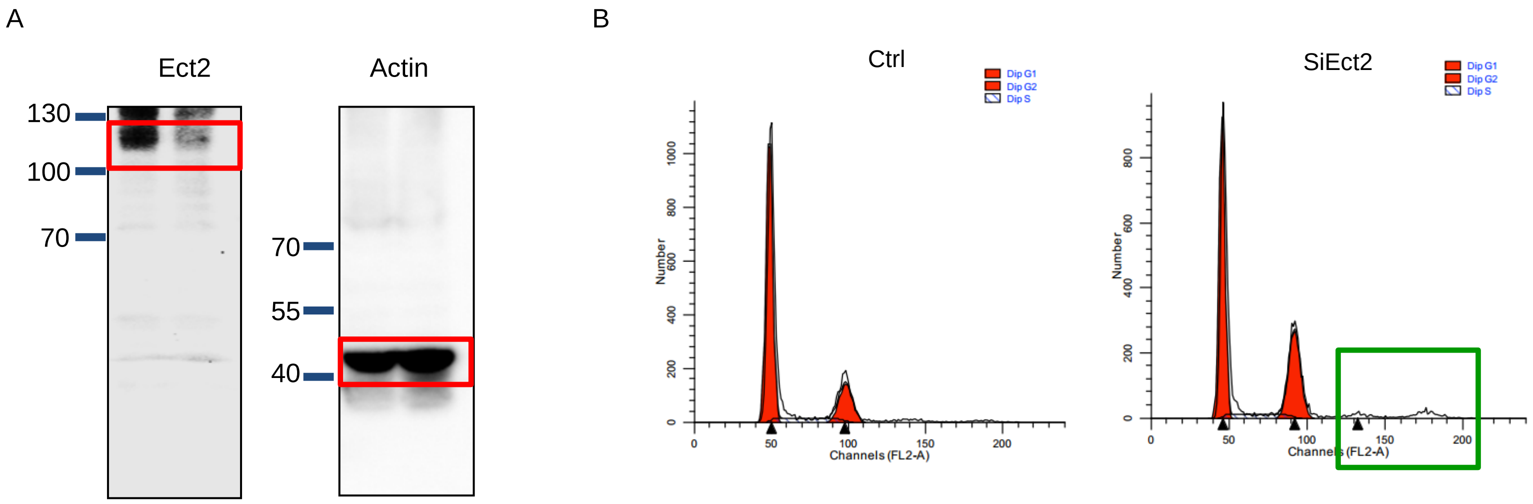

Supplementary Figures

Figure S3. The full length blots for Fig.3A.

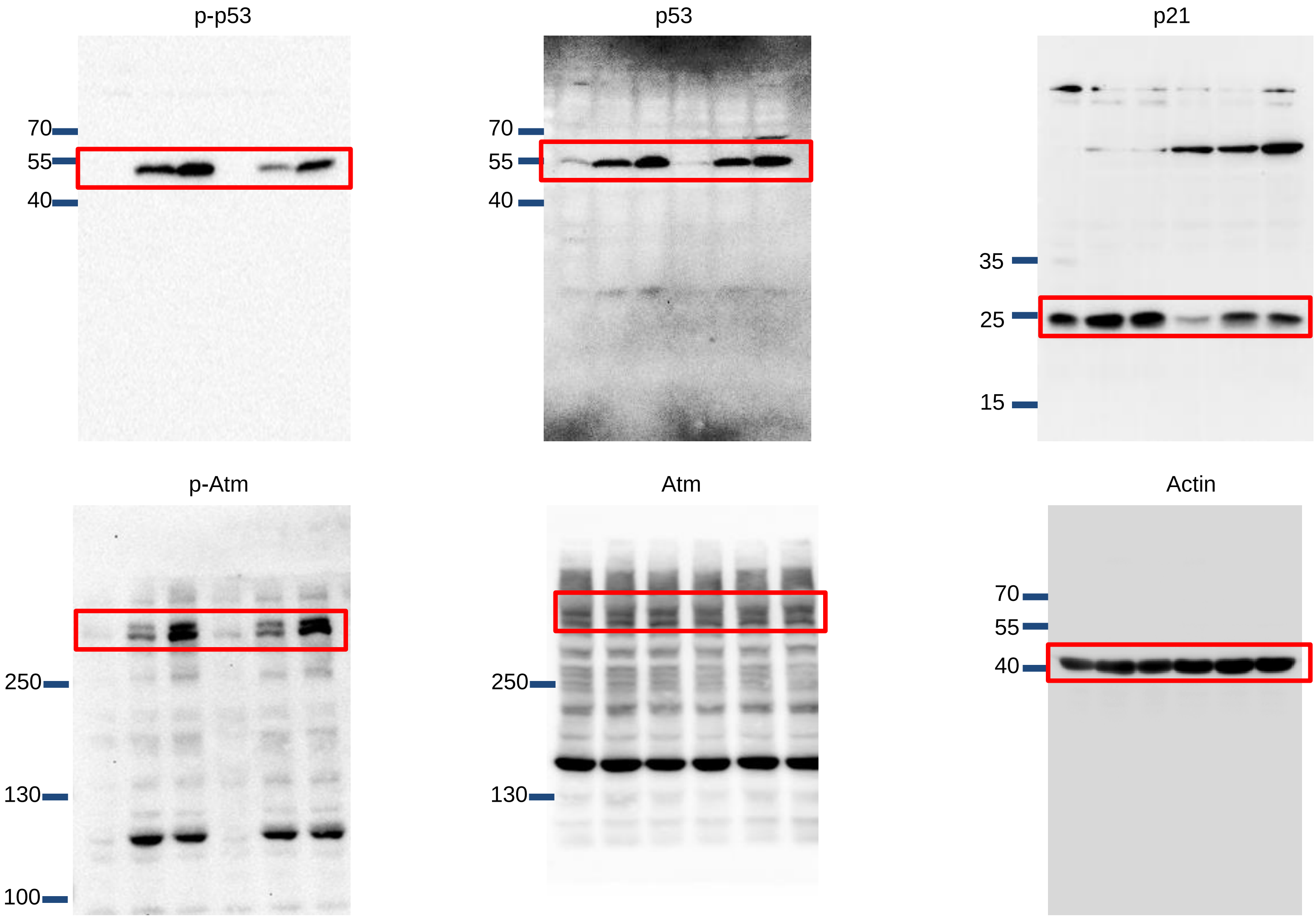

Supplementary Figures

Figure S4. Ect2 knockdown did not significantly affect cell survival in MEFs.

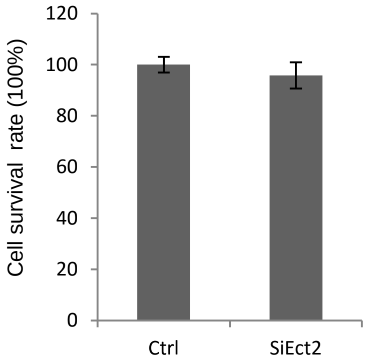

Supplementary Figures

Figure S5. A. Nocodazole and Dox treating for 4hrs. B. Nocodazole and Dox treating for 8hrs.

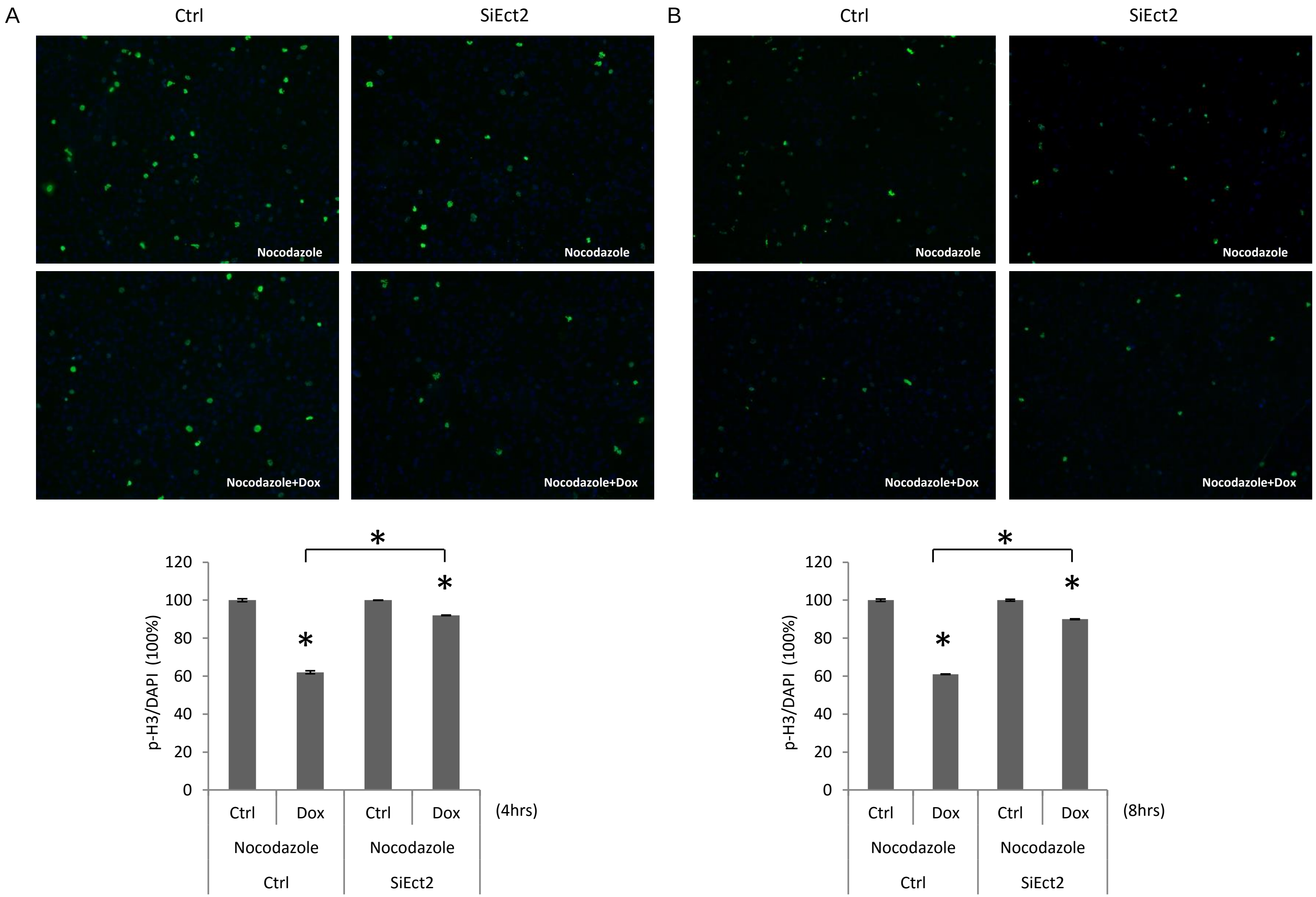

Supplementary Figures

Figure S6. The full length blots for Fig.6A.

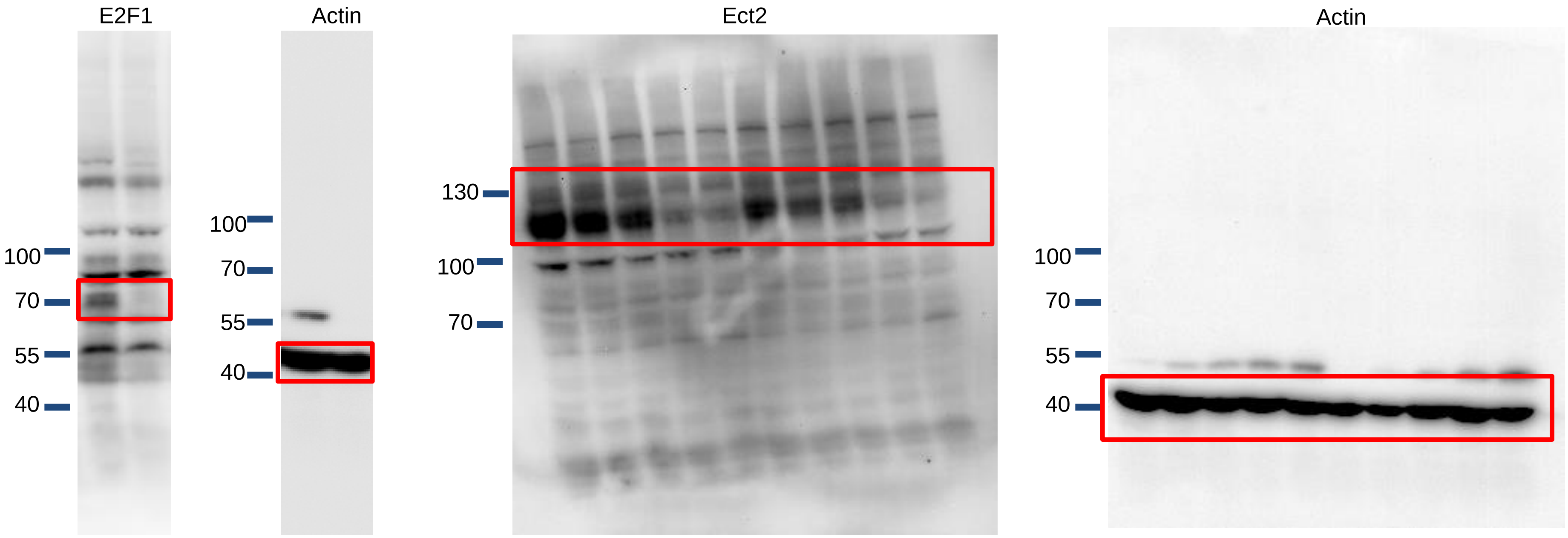

Supplementary Figures

Figure S7. The full length blots for Fig.6B.

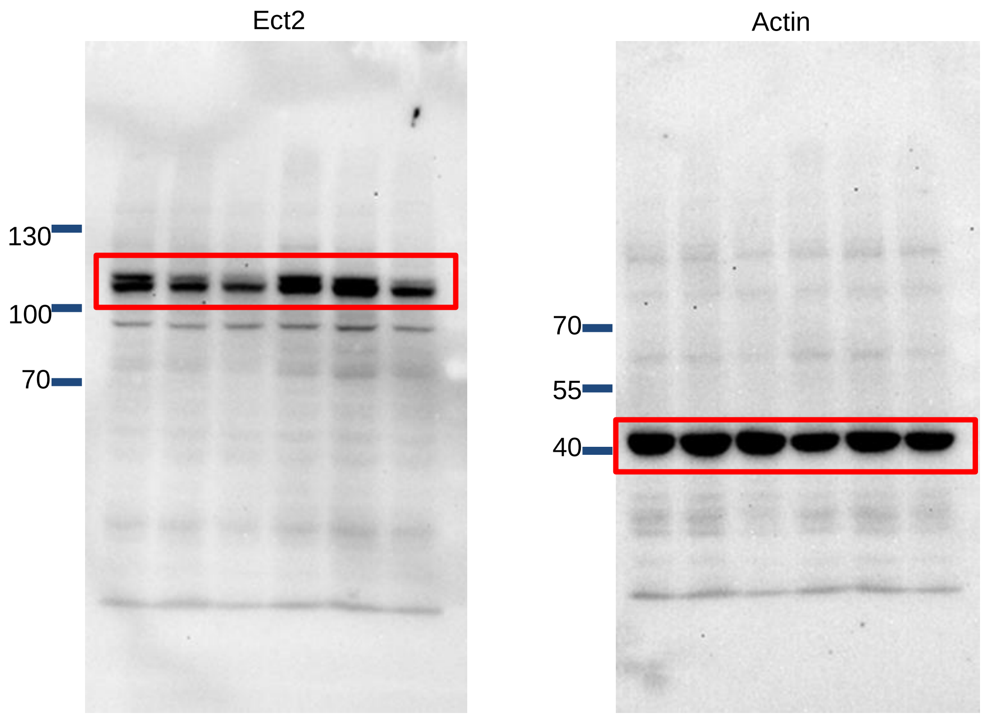

Supplementary Figures

Figure S8. The full length blots for Fig.6C.

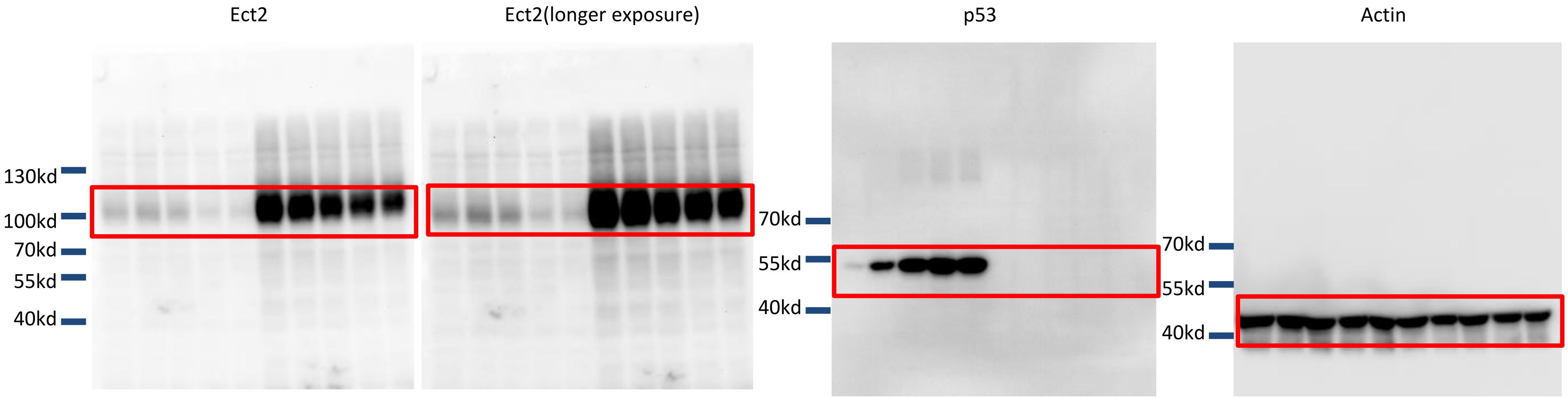

Supplementary Figures

Figure S9. The full length blots for Fig.7B.

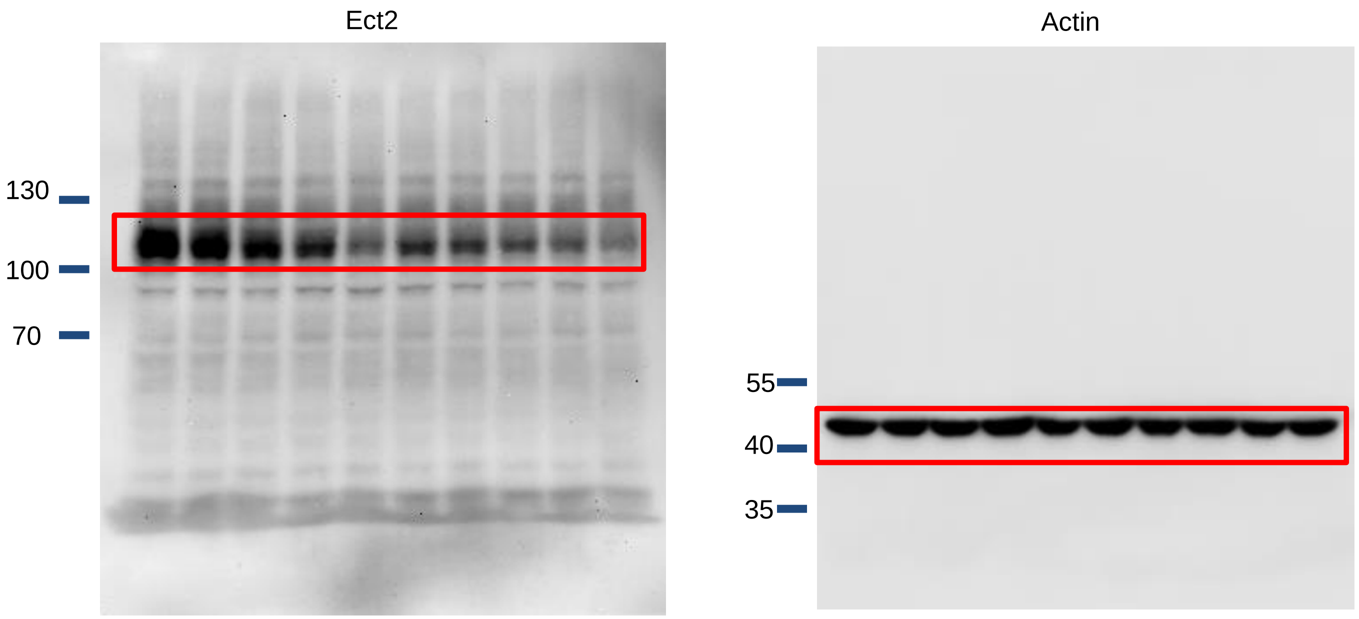

Supplementary Figures

Figure S10. The full length blots for Fig.7C.

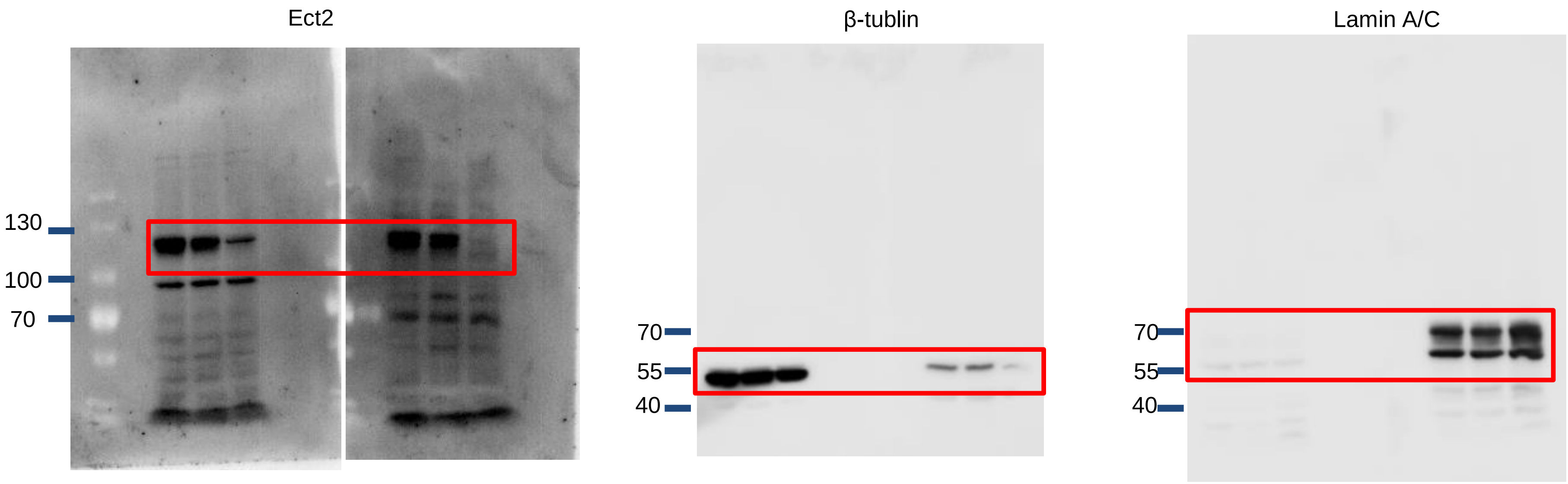

Supplement: Supplementary Information [file srep24504-s1.pdf]
